# Supplementary figures and images for: Aedes aegypti Hemocytes Mediate Antiviral Immunity
Source: Int J Mol Sci. 2025 Sep 9;26(18):8779. doi: 10.3390/ijms26188779 (PMC12470020; doi:10.3390/ijms26188779)

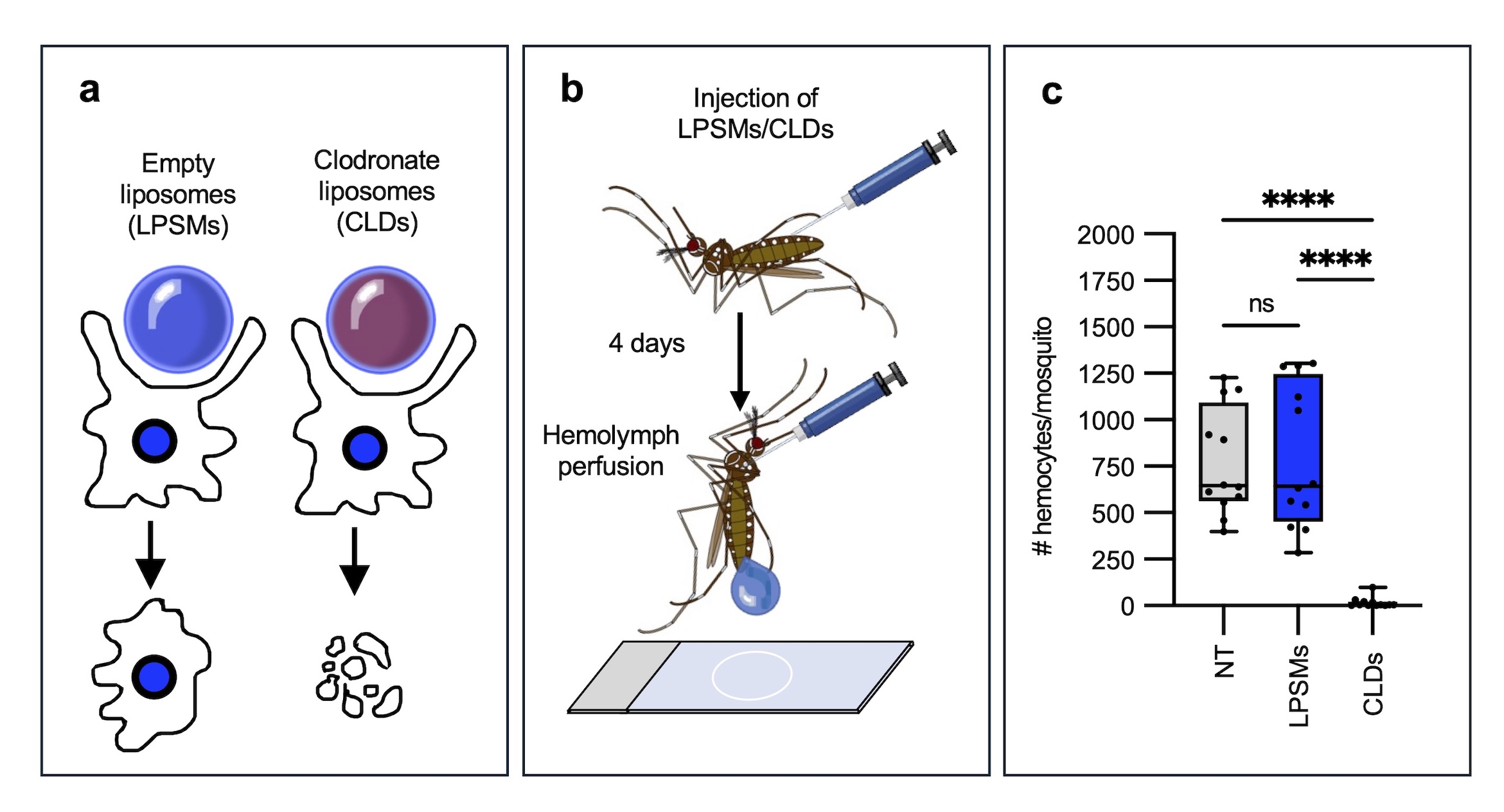

Supplement: Supplementary file 1 [file ijms-26-08779-s001.zip › Supplementary Figures/Supplementary Figure S1.jpg]

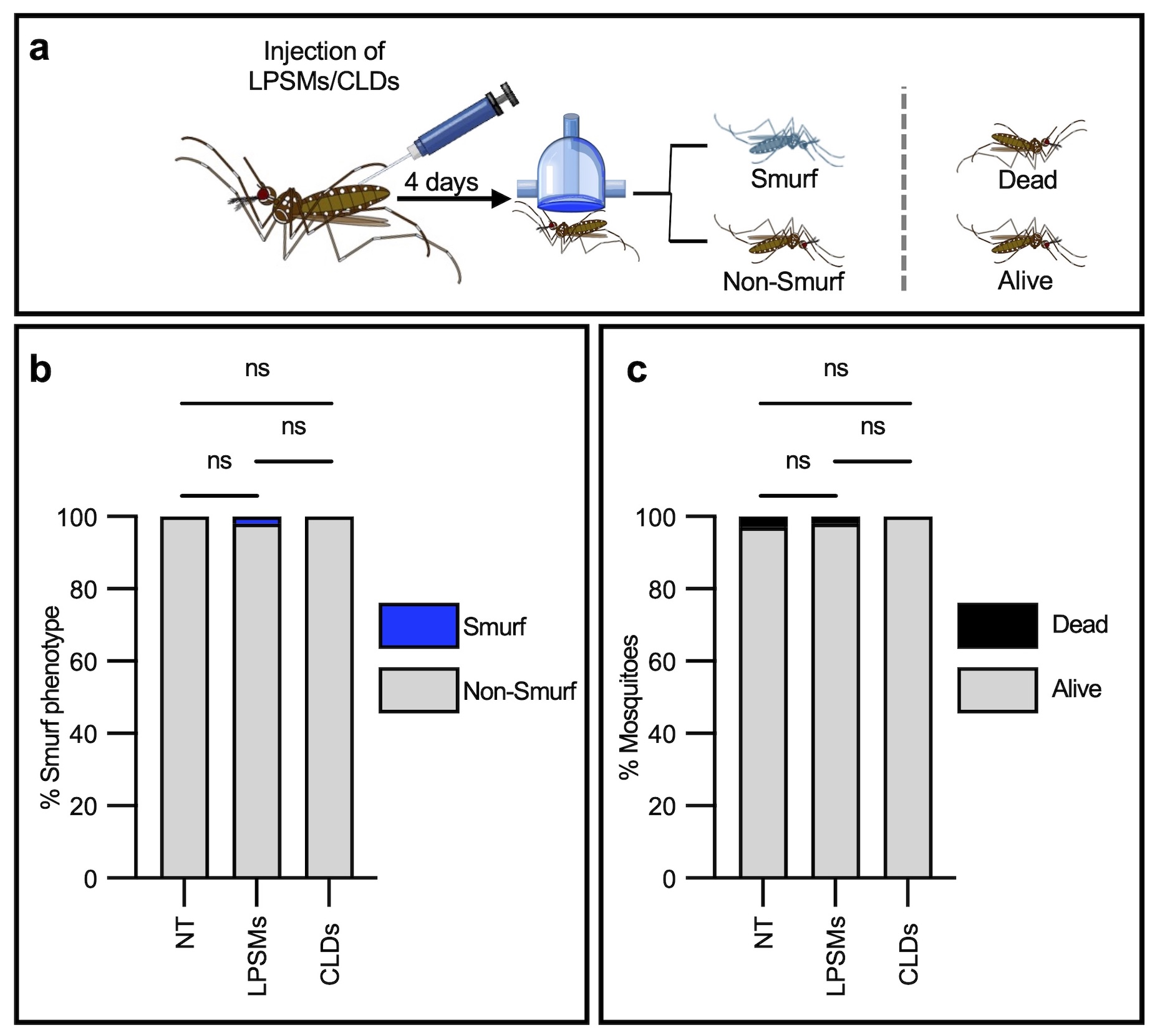

Supplement: Supplementary file 1 [file ijms-26-08779-s001.zip › Supplementary Figures/Supplementary Figure S2.jpg]

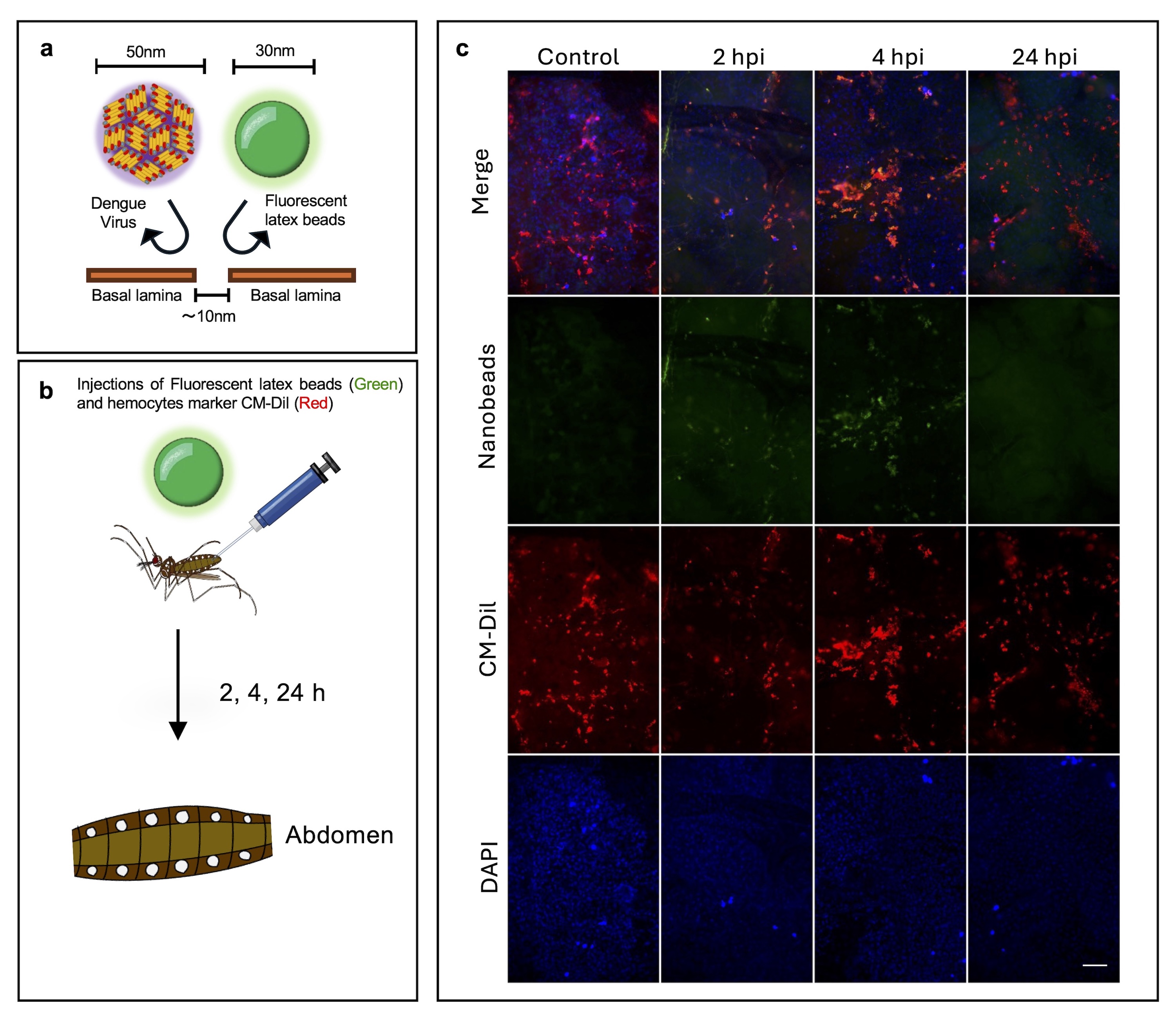

Supplement: Supplementary file 1 [file ijms-26-08779-s001.zip › Supplementary Figures/Supplementary Figure S3.jpg]

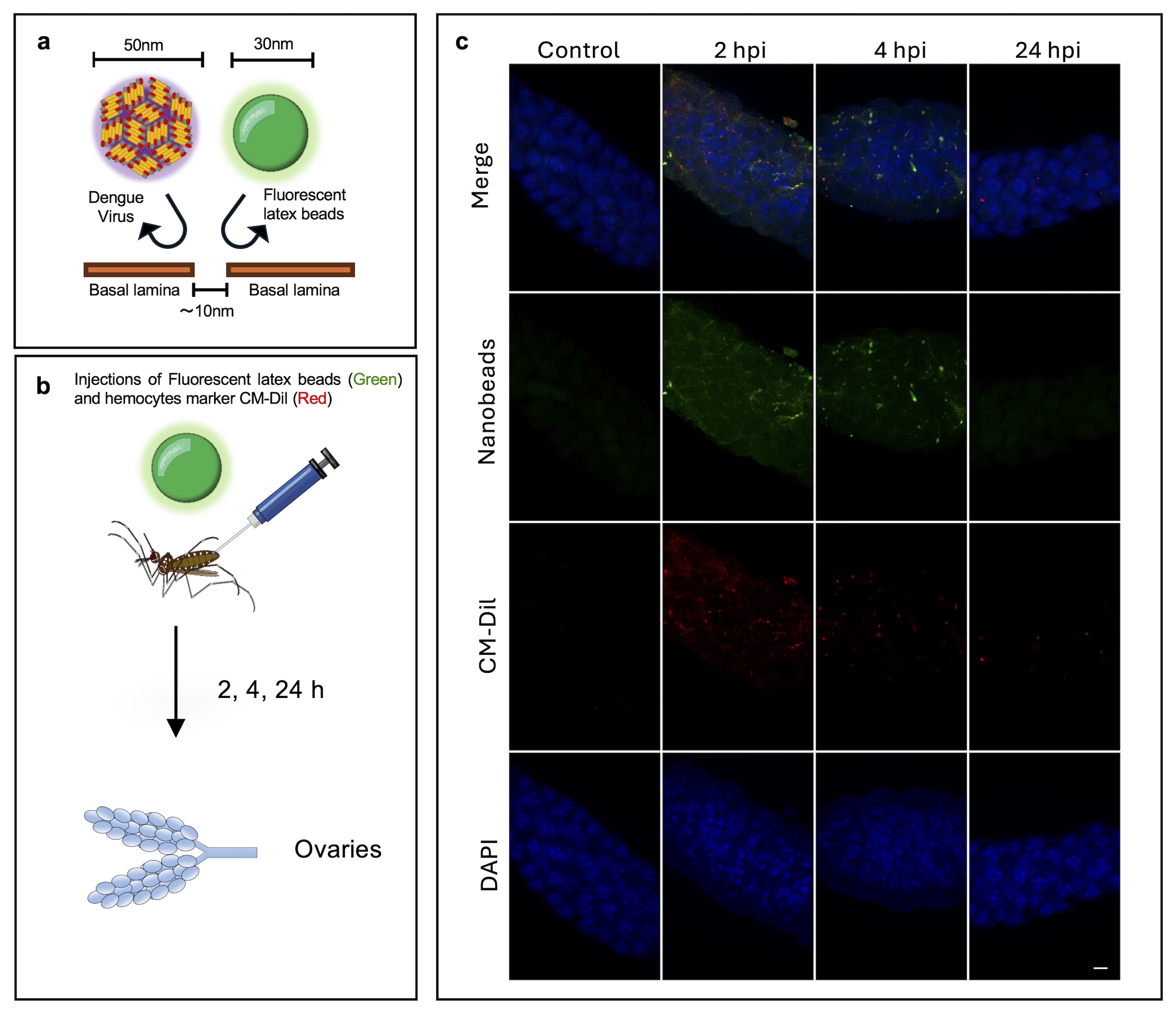

Supplement: Supplementary file 1 [file ijms-26-08779-s001.zip › Supplementary Figures/Supplementary Figure S4.jpg]

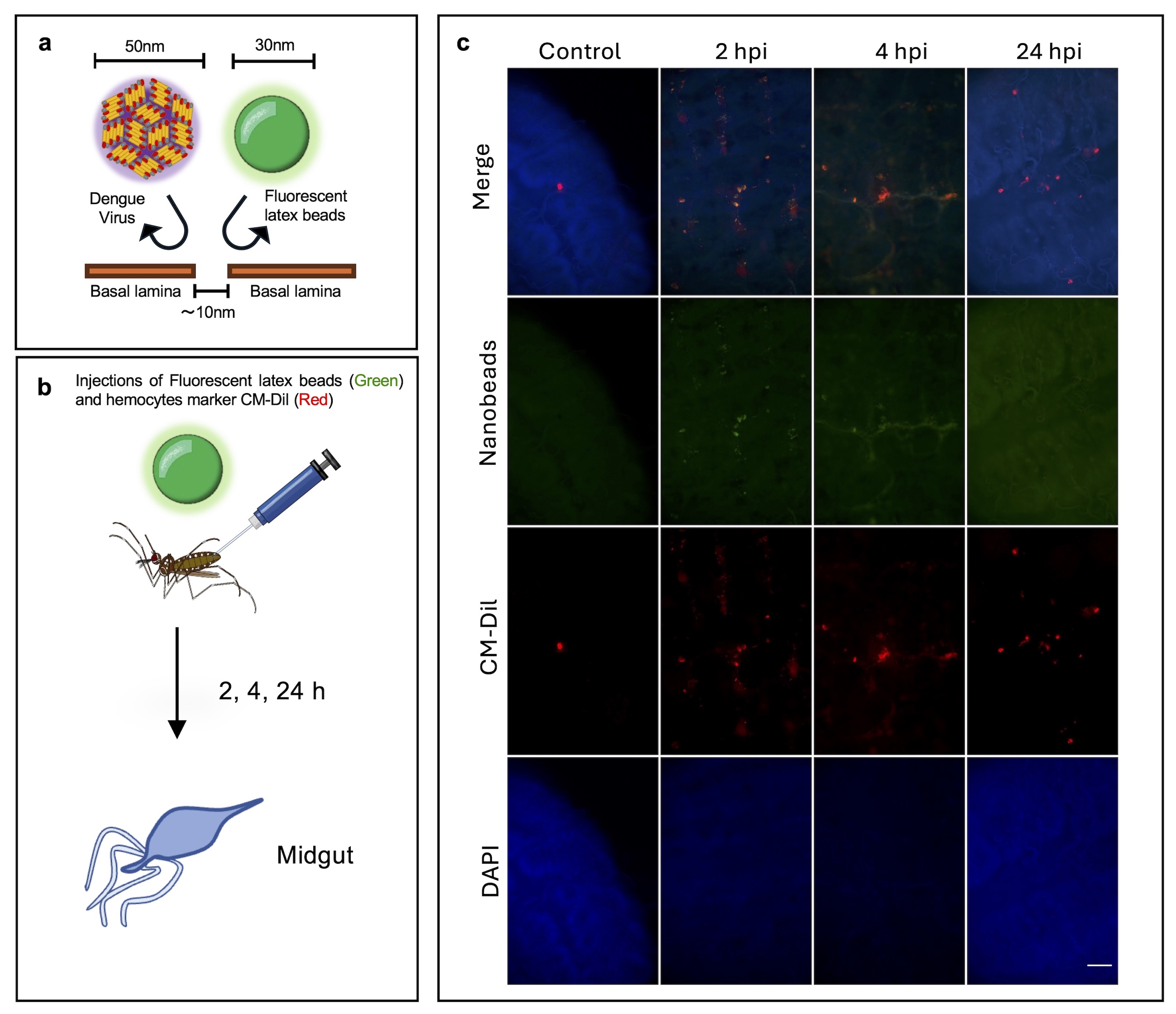

Supplement: Supplementary file 1 [file ijms-26-08779-s001.zip › Supplementary Figures/Supplementary Figure S5.jpg]
